# Single_cell_GRN: gene regulatory network identification based on supervised learning method and Single-cell RNA-seq data

**DOI:** 10.1186/s13040-022-00297-8

**Published:** 2022-06-11

**Authors:** Bin Yang, Wenzheng Bao, Baitong Chen, Dan Song

**Affiliations:** 1 School of Information Science and Engineering, Zaozhuang University, Zaozhuang, 277160 China; 2 School of Information and Electrical Engineering, Xuzhou University of Technology, Xuzhou, 221018 China; 3 Xuzhou First People’s Hospital, Xuzhou, 221000 China

**Keywords:** Single-cell, RAN-seq, Gene regulatory network, Supervised learning, Classification

## Abstract

Single-cell RNA-seq overcomes the shortcomings of conventional transcriptome sequencing technology and could provide a powerful tool for distinguishing the transcriptome characteristics of various cell types in biological tissues, and comprehensively revealing the heterogeneity of gene expression between cells. Many Intelligent Computing methods have been presented to infer gene regulatory network (GRN) with single-cell RNA-seq data. In this paper, we investigate the performances of seven classifiers including support vector machine (SVM), random forest (RF), Naive Bayesian (NB), GBDT, logical regression (LR), decision tree (DT) and K-Nearest Neighbor (KNN) for solving the binary classification problems of GRN inference with single-cell RNA-seq data (Single_cell_GRN). In SVM, three different kernel functions (linear, polynomial and radial basis function) are utilized, respectively. Three real single-cell RNA-seq datasets from mouse and human are utilized. The experiment results prove that in most cases supervised learning methods (SVM, RF, NB, GBDT, LR, DT and KNN) perform better than unsupervised learning method (GENIE3) in terms of AUC. SVM, RF and KNN have the better performances than other four classifiers. In SVM, linear and polynomial kernels are more fit to model single-cell RNA-seq data.

## Introduction

Human diseases, especially polygenetic genetic diseases, mainly including heart disease, hypertension, diabetes, asthma and cancer, are caused by the interaction of multiple gene loci and environmental factors [1–4]. Therefore, to construct gene regulatory network (GRN) and analyze regulatory mechanism have contributed to finding out the key network nodes, which could make an importance role in formulating new treatment plans and drug targets [5–8].

For gene regulatory network modeling, the existing learning methods could be divided into two categories: supervised learning and unsupervised learning [9]. Supervised learning methods could simulate problem of gene regulatory network recognition as classification problem. For a certain transcription factor (TF), genes could be divided into either TF-regulating genes or non-TF-regulating genes. The known regulation relationships are utilized to train the classifier and predict the unknown regulation relations. Due to the guidance with prior knowledge, supervised learning methods have been presented to infer GRN in the past decade. Wang et al. proposed a novel supervised inference method of GRN based on linear programming to infer the potential known transcription regulators [10]. Mordelet and Vert presented support vector machine (SVM) algorithm to solve binary classification problem of GRN [11]. Cerulo et al. solved the problem of unreasonable selection of negative samples [12]. Gillani investigated the performances of the different kernel functions of SVM for GRN inference and given the guidance about the research on supervised learning in the future [13]. Brouard et al. proposed a Markov Logic network to infer GRN and asymmetric bagging was utilized to handle the unbalanced training data [14]. Many neural network models have been also utilized to infer GRN [15–17].

The gene expression data used in supervised learning algorithms are all obtained by traditional sequencing technology, such as DNA microarray. However, biological tissue is composed of a variety of heterogeneous cells, and the differences between single cells may have a profound impact on the functions of multicellular organisms. In recent years, single-cell RNA-seq technology has been developed, which can be used for unbiased, repeatable, high-resolution and high-throughput transcription analysis of single cells [18–20]. Compared with the traditional transcriptome analysis of colony cells, single-cell RNA-seq technology can obtain the expression information of nearly 3000 genes in a single cell, which provides a powerful tool for distinguishing the transcriptome characteristics of various cell types in biological tissues, and comprehensively revealing the heterogeneity of gene expression between cells and the regulatory relationships between genes [21–24]. However, single-cell RNA-seq data has many shortcomings, such as high noise, many missing values, etc., so it is still challenging to reconstruct GRN using single-cell RNA-seq. Chan et al. proposed an information theory algorithm based on multivariate information measures to infer GRN according to single-cell data [25]. Nan et al. created time-stamped cross-sectional expression data and utilized regularized linear regression to identify GRN [26]. Matsumoto et al. proposed a novel GRN inference based on ordinary differential equation from single-cell RNA-seq [27].

In order to investigate the performances of supervised learning methods for GRN inference with single-cell RNA-seq data, we proposed a hybrid supervised learning method (Single_cell_GRN), which utilizes SVM, random forest, Naive Bayesian (NB), GBDT, logical regression (LR), decision tree (DT) and K-Nearest Neighbor (KNN) to infer gene regulatory network separately. For SVM, linear kernel, polynomial kernel and radial basis function are utilized and investigated. Three real single-cell RNA-seq datasets from mouse and human are utilized to test the supervised learning methods.

## Methods

### Supervised learning methods

#### Support vector machine

Support vector machine is a kind of machine learning method based on statistical theory, which was proposed by Vapnik [28]. It is mainly utilized to solve two-class classification problems. The main idea is to map the samples to the high-dimensional feature space (kernel space), in which the linear classifier is constructed in order to obtain the largest interval [29]. Due to its advantages in solving small samples, and nonlinear and high-dimensional pattern recognition, SVM has been widely applied to text classification [30], bioinformatics [31, 32], financial data prediction [33], signal processing [34] and image processing [35].

The mechanism of SVM is to search the optimal hyperplane to meet the classification requirements. Two restricted conditions need to be considered, such as classification accuracy and maximizing the blank area on both sides of the hyperplane. So the learning process of SVM is an optimization problem.

Give the training dataset (*x*_*i*_, *y*_*i*_), *i = *1,2,*…*,*N*, *N* is the number of data, *x*_*i*_ is feature vector, and *y*_*i*_ is classification label (+1, –1) . Hyperplane is labeled as (*w*·*x*) + *b* = 0) *w* and *b* are coefficients and deviation term). The optimal hyperplane problem is constructed as follows.

1 $$\begin{gathered} \mathop {\min }\limits_{\alpha } \frac{1}{2}\sum\limits_{i = 1}^{N} {\sum\limits_{j = 1}^{N} {\alpha_{i} \alpha_{j} y_{i} y_{j} (x_{i} \cdot x_{j} )} - \sum\limits_{i = 1}^{N} {\alpha_{i} } } \hfill \\ s.t.\;\;\;\;\;\sum\limits_{i = 1}^{N} {\alpha_{i} y_{i} } = 0 \hfill \\ \;\;\;\;\;\;\;\;\;\alpha_{i} \ge 0 \hfill \\ \end{gathered}$$

By solving the optimal problem, the optimal solution $\alpha^{*} = (\alpha {}_{1}^{*} ,\;\alpha {}_{2}^{*} , \ldots ,\;\alpha {}_{N}^{*} )^{T}$ is obtained. The optimal classification function could be also obtained as follows.

2 $$f(x) = {\text{sgn}} \{ \sum\limits_{i = 1}^{N} {\alpha_{i}^{*} y_{i} (x_{i} \cdot x)} + b^{*} \} .$$

Linear SVM utilizes hyperplane to divide two kinds of data. If the data itself is nonlinear, it is not suitable to use hyperplane as decision boundary. By kernel function, SVM can be applied to solve nonlinear classification problems. Kernel function is utilized to replace the inner product between two instances after a nonlinear transformation. The common kernel functions contain linear kernel, polynomial kernel and radial basis function (rbf), which are defined as followed.

3 $$K_{linear} (x,y) = x \cdot y.$$

4 $$K_{polynomial} (x,y) = ((x \cdot y) + 1)^{d} .$$

5 $$K_{rbf} (x,y) = \exp ( - \gamma \left\| {x - y} \right\|^{2} ).$$

#### Random forest

Random forest (RF) is a flexible and easy-to-use machine learning algorithm [36]. Compared with SVM, the selection of super parameters has less effect on the performance of RF, which is commonly utilized to solve classification and regression problems [37–40]. RF was proposed based on ensemble learning method and decision tree. Its basic unit is decision tree, which is also a classifier. For an input sample, *N* trees could create *N* classification results. RF integrates all the classification results by voting method and specifies the category with the most voting times as the final output. The principle of RF is given as follows.

1. Firstly *M* samples are randomly selected from the sample set by bootstrap algorithm. For each sample, *K* features are selected randomly from all attributes. According to the selected *K* features, a decision tree is established.
2. Repeat step (1) *N* times in order to obtain *N* decision trees.
3. Input variables are given to each decision tree, which could output a result. *N* decision trees could get *N* classification results.
4. Calculate the number of votes of all classes and select the classification result with the highest number of votes as the final category.

#### Naive Bayesian

Naive Bayesian is built on Bayes' theorem and is a typical generative learning method [41]. The main idea is to adopt the attribute conditional independence assumption. It assumes that all attributes are independent of each other, and the impact of different attributes on the classification results is irrelevant. The algorithm can not only simplify the calculation and be easy to implement, but also has good robustness. It is commonly used in statistical decision-making fields such as text document classification [42] and medical diagnosis [43].

Bayesian theorem is expressed as follows:

6 $$P{(}c|x{)} = \frac{{P{(}c{)}P{(}x|c{)}}}{{P{(}x{)}}}.$$

where *c* denotes class, *P*(*c*) represents a priori probability, *P*(*c *| *x*) indicates a posteriori probability, *P*(*x *| *c*) denotes the class conditional probability and *P*(*x*) is the edge probability of *x*.

Based on the assumption of attribute conditional independence, Eqs. (6) can be rewritten as:

7 $$P{(}x|c{)} = \frac{{P{(}c{)}}}{{P{(}x{)}}}\prod\limits_{i = 1}^{d} {P{(}x_{i} |c{)}} .$$

where *d* is the number of attributes and *x*_*i*_ is the value on the *i*-th attribute.

Because the denominator in Eqs. 7 is the same for all categories, it has no impact on the result. Therefore, the simplified formula of Naive Bayes is defined as follows.

8 $$c{(}x{)} = \mathop {argmax}\limits_{c \in C} P{(}c{)}\prod\limits_{{i = {1}}}^{d} {P{(}x_{i} |c{)}} .$$

#### GBDT

GBDT is a type of ensemble learning method [44] and an algorithm with strong generalization ability [45, 46]. The main idea is to use the negative gradient of the loss function to simulate the residual, and take the residual of the previous tree as the input of the next tree. In each iteration, the loss decreases rapidly along the negative gradient direction, and finally accumulates the prediction results of all trees as the final result of the model.

The training dataset is *T*= {(*x*_1_,*y*_1_),(*x*_2_,*y*_2_),^…^, (*x*_N_,y_N_)}, and the loss function is *L*(*y*, *f*(*x*)) , where *xi* represents the feature vector and *y* is the label. The main flowchart of the algorithm is indicated as follows.

(1) Initialize weak learner.

9 $$f_{0} (x) = \mathop {\arg \min }\limits_{c} \sum\limits_{i = 1}^{N} {L(} y_{i} ,c).$$

(2) For *m*= 1,2,^…^,*M*, where *M *is the number of iterations.

- Calculate the negative gradient of the loss function in the current tree, the residual is written as:
  10 $$r_{mi} = - [\frac{{\partial L{(}y_{i} {,}\;f{(}x_{i} {))}}}{{\partial f{(}x_{i} {)}}}]_{{f(x) = f_{{m - {1}}} (x)}} .$$
- Fit a regression tree to the target $r_{mi}$ and compute the leaf node region $R_{mj} \;(j = {1,2,} \cdots ,J)$ of the regression tree.
- For $j = {1,2,} \cdots ,J$, the optimal coefficient of leaf node region is calculated.
  11 $$c_{mj} = \mathop {argmin}\limits_{c} \sum\limits_{{x_{i} \in R_{mj} }} {L{(}y_{i} ,f_{{m - {1}}} {(}x_{i} ) + c{)}} .$$
- The strong learner in this iteration is obtained.
  12 $$f_{m} (x) = f_{m - 1} (x) + \sum\limits_{J}^{j = 1} {c_{mj} } I{(}x \in R_{mj} {)}{\text{.}}$$

(3) After all the iterations, the strong learner is obtained.

13 $$f_{M} (x){ = }\sum\limits_{m = 1}^{M} {\sum\limits_{{j{ = }1}}^{J} {c_{mj} } } I(x \in R_{mj} ).$$

#### Logical regression

Logistic regression [47] is an important statistical model in machine learning and has been widely used in biology, epidemiology and other fields [48, 49]. Logical regression consists of linear regression and Sigmoid function. The continuous values of the regression results are allocated between 0 and 1 in order to solve the classification problems. The specific process is as follows.

(1) Firstly, assuming that *x *is the input vector, *θ* is the parameters to be solved, and *y* represents the prediction result of linear regression, the linear regression model is given as follows.

14 $$y = \theta_{0} + \theta_{{1}} x_{{1}} + ... + \theta_{n} x_{n} = \sum\limits_{{i = {1}}}^{n} {\theta_{i} x_{i} } = \theta^{T} x.$$

(2) In LR, the logic function is Sigmoid function, which is defined as follows.

15 $$g(z) = \frac{{1}}{{{1} + e^{ - z} }}.$$

According to Sigmoid function, the output of model is constructed as follows.

16 $$h_{\theta } (x){ = }\frac{1}{{1{ + }e^{{ - \theta^{T} x}} }}$$

The parameter $\theta$ can be estimated by the maximum likelihood method, and the final model can be obtained by continuously optimizing the parameters through inputting the test samples.

#### Decision tree

Decision tree is a machine learning method used to solve classification problems [50]. It is a tree structure that divides the data by making a series of decisions. A decision tree contains root node, internal nodes and leaf nodes. The decision-making process of the decision tree starts from the root node. By testing the corresponding characteristic attributes of the items to be classified, the output branches are selected according to the results. The generation of decision tree is a recursive process. Each step will pick up the optimal selection of the current state until the leaf node has been selected. Finally take the category stored in the leaf node as the final decision result.

In the generation process of decision tree, the key step is the measurement of feature selection. The feature selection is based on the principle that the samples contained in the branch nodes belong to the same category as much as possible. At present, there are three main algorithms for the construction of a decision tree, namely ID3, C4.5 and CART. In this paper, we select CART algorithm [51, 52].

CART algorithm utilizes binary recursive segment method to divide the sample set into two sub sample sets, which contains feature selection and tree pruning. *Gini* index is utilized to select the features, determine the optimal partition points and measure the purity of dataset *D*, which is defined as follows.

17 $$Gini{(}D{)} = \sum\limits_{{i = {1}}}^{n} {p{(}x_{i} {)}} {(1} - p{(}x_{i} {)) = 1 - }\sum\limits_{{i = {1}}}^{n} {p{(}x_{i} {)}^{2} } .$$

where *p*(*x*_*i*_) is the probability of category *x*_*i*_ , and *n* is the number of categories in *D*. *Gini* (*D*) reflects the probability that the category labels of two samples are inconsistent, which are randomly selected from dataset *D*. Therefore, the smaller the *Gini* (*D*) is, the higher the purity of the dataset *D* is.

#### K-Nearest Neighbor

K-Nearest Neighbor is a commonly used machine learning algorithm [53, 54]. It can be utilized to solve classification and regression problems, and is widely used in data mining and pattern recognition. The algorithm idea is to identify the *K* training samples of the known categories that are most similar to the test sample based on some distance measures in the sample space. Then judge the category of the sample based on the information of *K* neighbors. The main algorithm flowchart of KNN is given as follows.

- (1) Build the training sample set and calculate the distances between the test sample and the training samples based on the distance measurement.
- (2) Sort the training samples in ascending order according to the distances.
- (3) Select the *K* training samples closest to the test one as the *K* neighbors of the test sample.
- (4) Count the category frequencies of *K* neighbors, and select the category with the highest frequency as the category of the test sample.

### GRN inference with single-cell RNA-seq data and supervised learning method

**Fig. 1:**
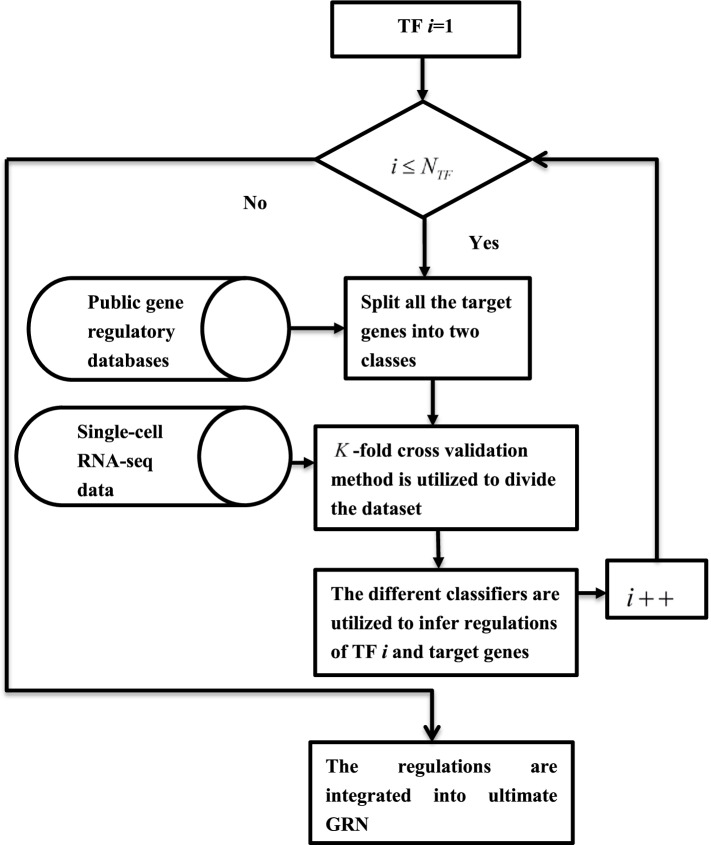
The flowchart of gene regulatory network inference with single-cell RNA-seq data and supervised learning method

For the inference of gene regulatory networks, the complex regulatory relationships among genes are identified, which could be evolved to two-class problems. Single-cell RNA-seq data and the corresponding regulatory relationships between genes are collected from the public databases. Count up the number of TF as *N*_*TF*_. With the regulatory networks verified by biology experiments from the well-known databases, for each regulatory factor *i*, all the target genes set can be divided into two categories. The target genes regulated by the regulator factor *i* are marked as positive gene set, while the target genes not regulated by the regulator factor *i* are marked as negative gene set. The single-cell RNA-seq data of two kinds of gene sets are constructed. *K*-fold cross validation method is utilized to divide the training and testing datasets in order infer the regulatory relationships between all target genes and regulatory factors. For each classification problem, different classification methods are selected. If the number of positive samples is zero, the sample is classified as negative, which reveal that there are no regulatory relationships between the regulator factor *i* and the targets. Otherwise SVM, RF, NB, GBDT, LR, DT and KNN are utilized, respectively. When the regulations of all regulatory factors have been inferred, the algorithm stop; otherwise repeat the above process. The regulations of all TFs are integrated in order to obtain the overall GRN. The flowchart is depicted in Fig. 1.

## Experiments and discussions

Three real single-cell RNA-seq datasets are utilized to test our methods. The first dataset is derived from primitive endoderm cells differentiated from mouse embryonic stem cells, which includes 456 cells (Data1) [55]. The second dataset was derived from scRNA-Seq data obtained to examine direct reprogramming from mouse embryonic fibroblast (MEF) cells to myocytes, which includes 405 cells (Data2) [56]. The third dataset was derived from definitive endoderm (DE) cells differentiated from human ES cells, which includes 758 cells (Data3) [57]. Three extracted sub networks and the validation regulatory relationships are from the previous study [27].

Receiver Operating Characteristic (ROC) curve considers true positive rate (TPR) and false positive rate (FPR), and could accurately reflect the relationship between TPR and FPR of a learner, which is a comprehensive manner to evaluate model sensitivity and specificity. TPR denotes the proportion of the inferred real regulatory relationships in all real regulations. FPR represents the proportion of the inferred false-positive regulatory relationships in all the true non-regulations. Area Under ROC Curve (AUC) is the area covered by ROC curve, which could reflect the performance of the learner more intuitively. In this part, ROC curves and AUC are utilized to evaluate our methods.

### Results

**Fig. 2:**
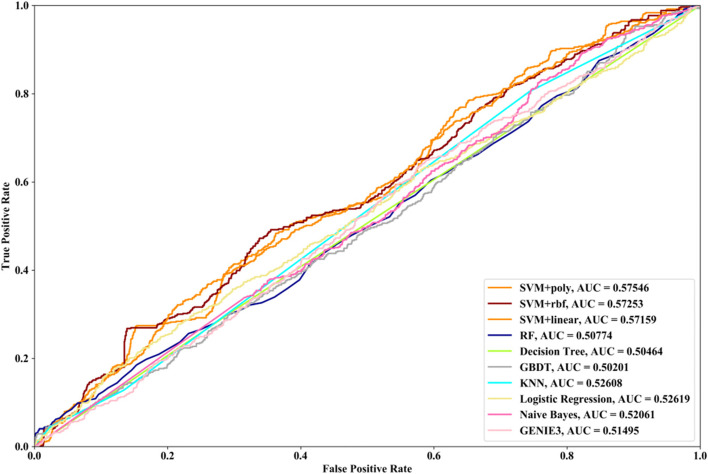
AUC and ROC performances of ten methods by LOOCV with Data1 for GRN inference

**Fig. 3:**
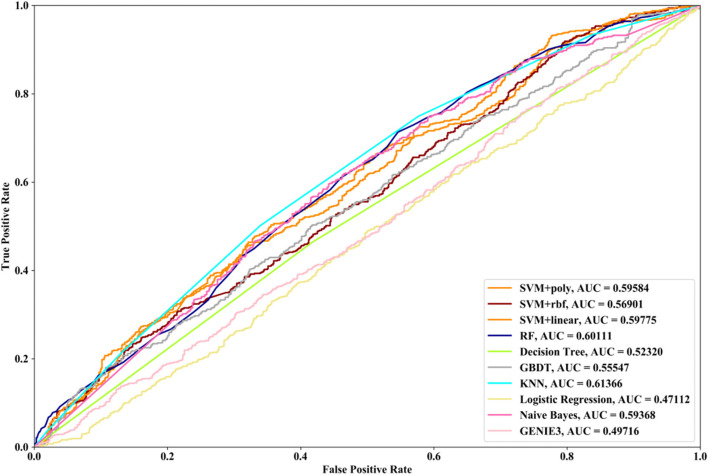
AUC and ROC performances of ten methods by LOOCV with Data2 for GRN inference

**Fig. 4:**
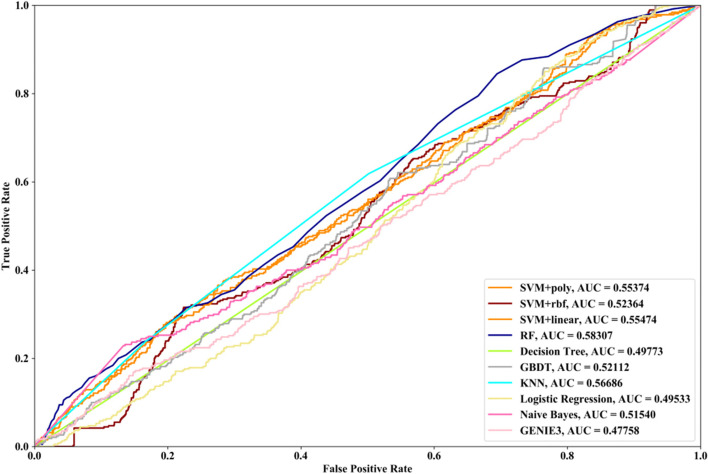
AUC and ROC performances of ten methods by LOOCV with Data3 for GRN inference

In this part SVM with different kernel functions (linear kernel (SVM + linear), polynomial kernel (SVM + poly) and radial basis function (SVM + rbf)), RF, NB, GBDT, LR, DT and KNN are utilized to infer GRN with three real single-cell RNA-seq data, respectively. Leave-One-Out Cross Validation (LOOCV) is utilized to classify the unknown regulatory relationships. To better evaluate the performance of supervised learning algorithms, the famous unsupervised learning method (GENIE3) is also utilized to infer the same GRNs, which has the highest performance in the DREAM3 Challenge. The ROC curves and the corresponding AUC values of ten methods are depicted in Figs. 2, 3 and 4, respectively. For Data1, SVM with polynomial kernel has the highest AUC value, which is 0.5% higher than SVM + rbf, 0.7% higher than SVM + linear, 13.3% higher than RF, 14% higher than DT, 14.6% higher than GBDT, 9.4% higher than KNN, 9.5% higher than LR, 10.5% higher than NB and 11.8% higher than GENIE3. From the results of ROC and AUC, SVM methods with three different kernel functions perform better than RF, DT, GBDT, KNN, LR and NB. GENIE3 performs better than RF, DT and GBDT, worse than other six classifiers. For Data2, in terms of ROC curve, KNN and RF have the similar performances, which are better than other eight methods. In terms of AUC, KNN has the best performance, which is 2.9% higher than SVM + poly, 7.8% higher than SVM + rbf, 2.7% higher than SVM + linear, 2.1% higher than RF, 17.3% higher than DT, 10.5% higher than GBDT, 30.3% higher than LR, 7.2% higher than NB and 23.4% higher than GENIE3. Unsupervised learning method (GENIE3) and LR have lower AUC value than other eight supervised learning methods, which are less than 0.5. For Data3, RF has the highest AUC value, which is 5.3% higher than SVM + poly, 5.1% higher than SVM + linear, 11.3% higher than SVM + rbf, 17.1% higher than DT, 11.9% higher than GBDT, 2.9% higher than KNN, 17.7% higher than LR, 13.1% higher than NB, and 22.1% higher than GENIE3. GENIE3 has the worst performance. Combined with ROC curves, we can see that in most cases the performance GENIE3 is less than random and supervised learning methods perform better than unsupervised learning method.

### Discussions

**Fig. 5:**
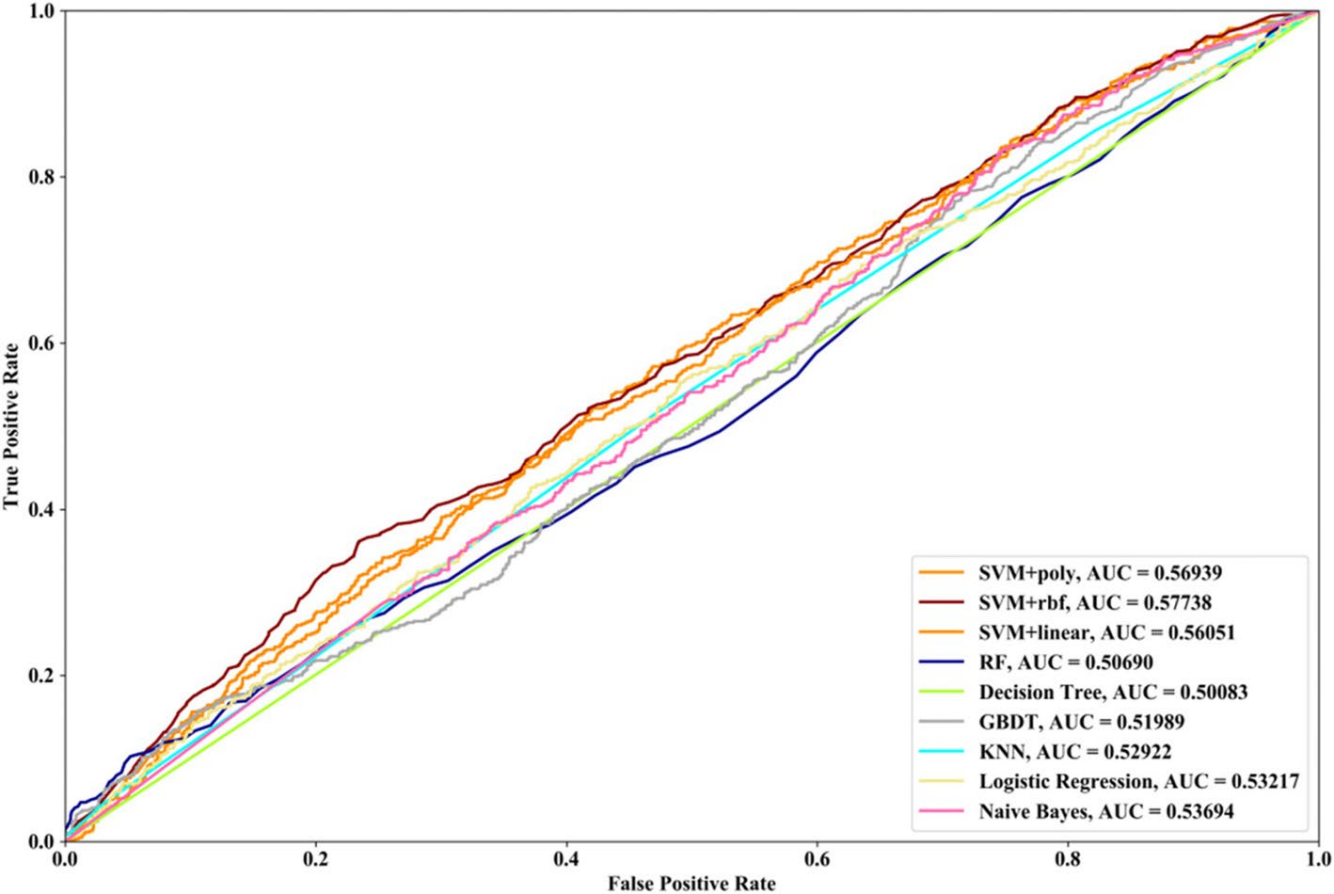
AUC and ROC performances of nine classifiers with Data1 and 3-cross validation method for GRN inference

**Fig. 6:**
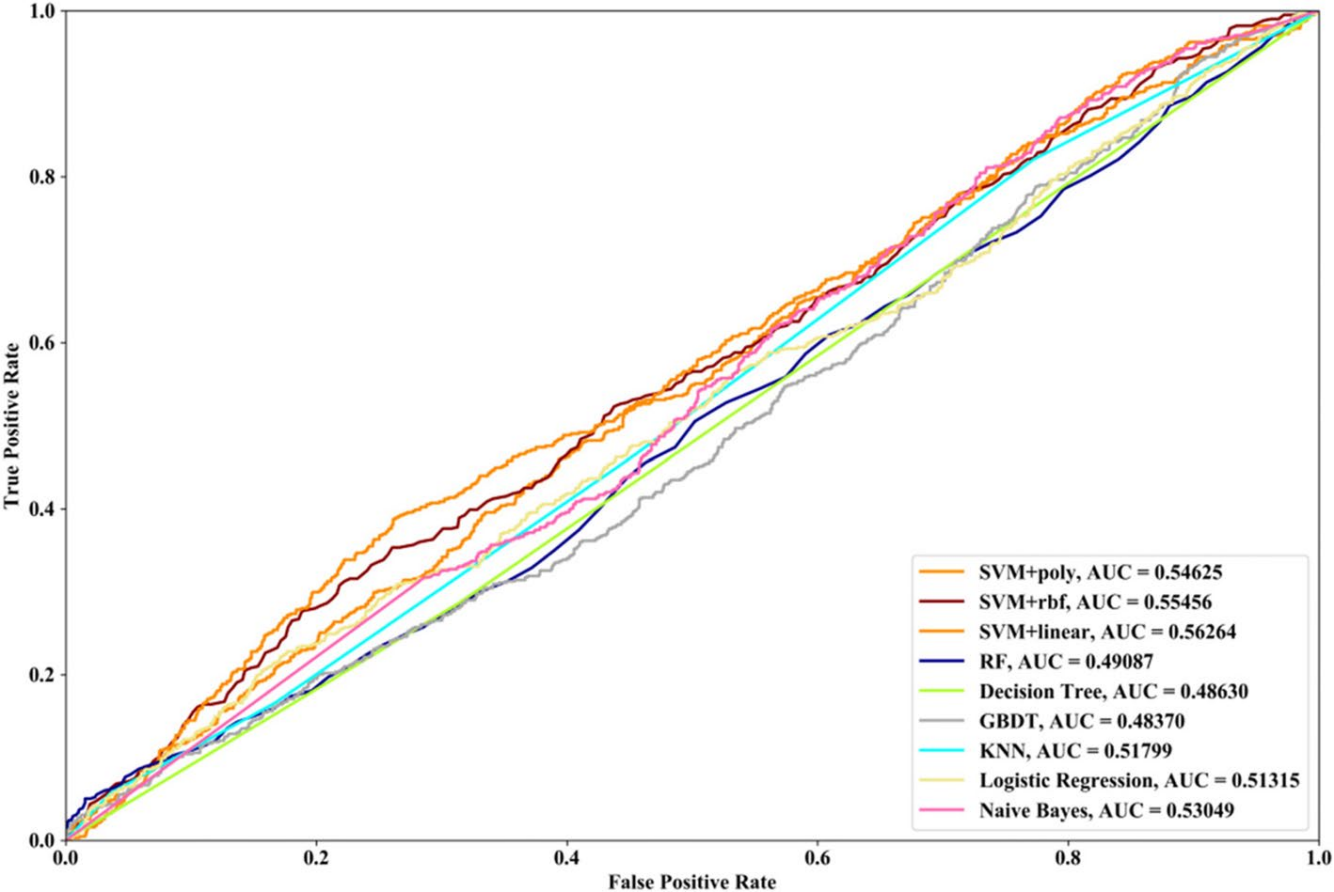
AUC and ROC performances of nine classifiers with Data1 and 5-cross validation method for GRN inference

**Fig. 7:**
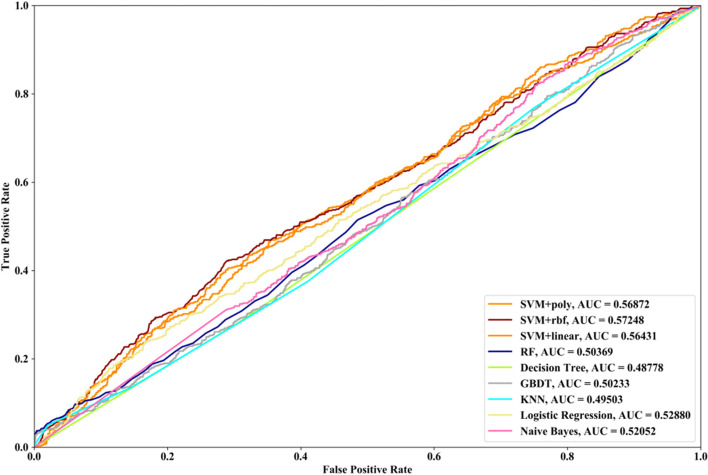
AUC and ROC performances of nine classifiers with Data1 and 10-cross validation method for GRN inference

**Fig. 8:**
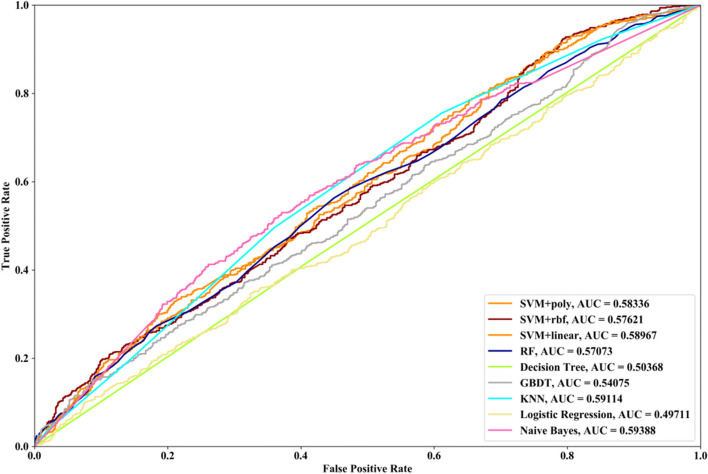
AUC and ROC performances of nine classifiers with Data2 and 3-cross validation method for GRN inference

**Fig. 9:**
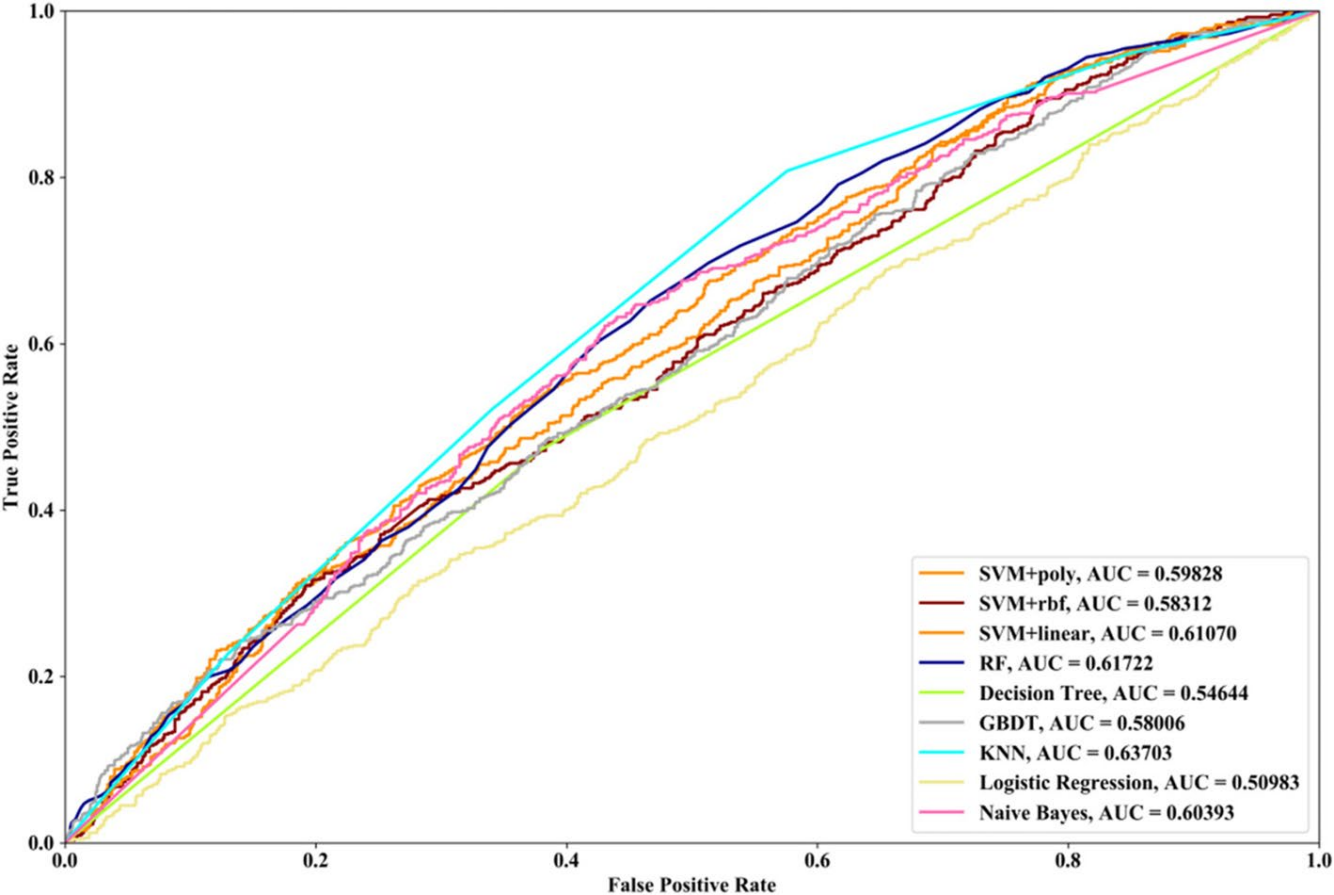
AUC and ROC performances of nine classifiers with Data2 and 5-cross validation method for GRN inference

**Fig. 10:**
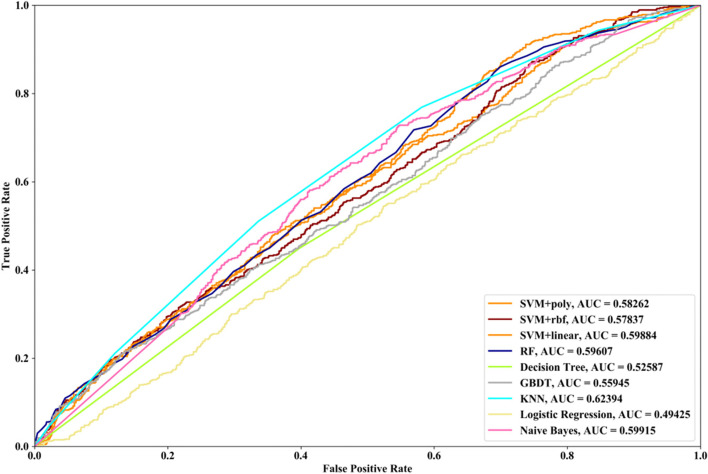
AUC and ROC performances of nine classifiers with Data2 and 10-cross validation method for GRN inference

**Fig. 11:**
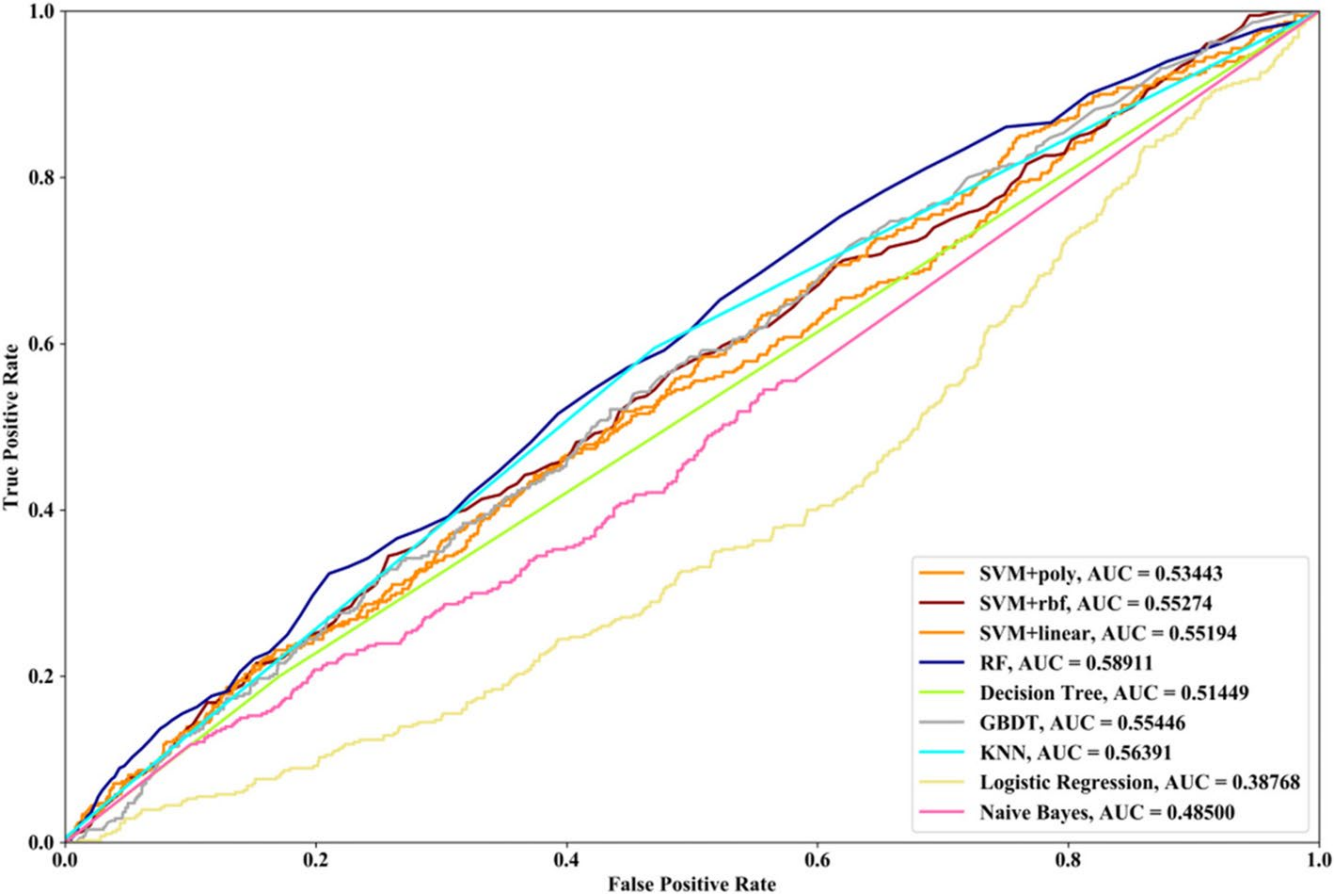
AUC and ROC performances of nine classifiers with Data3 and 3-cross validation method. for GRN inference

**Fig. 12:**
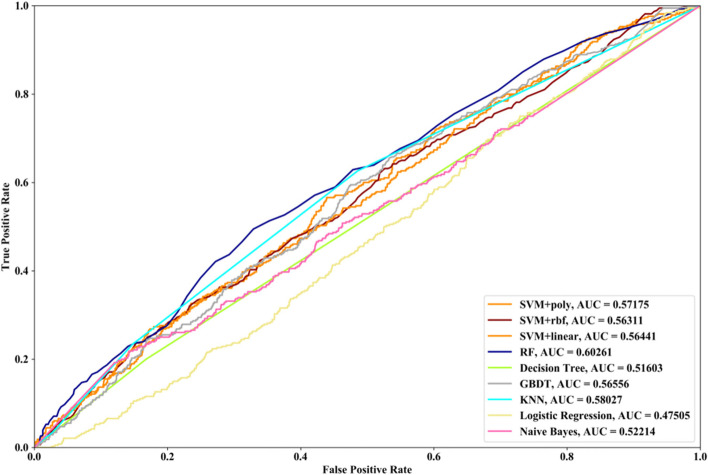
AUC and ROC performances of nine classifiers with Data3 and 5-cross validation method for GRN inference

**Fig. 13:**
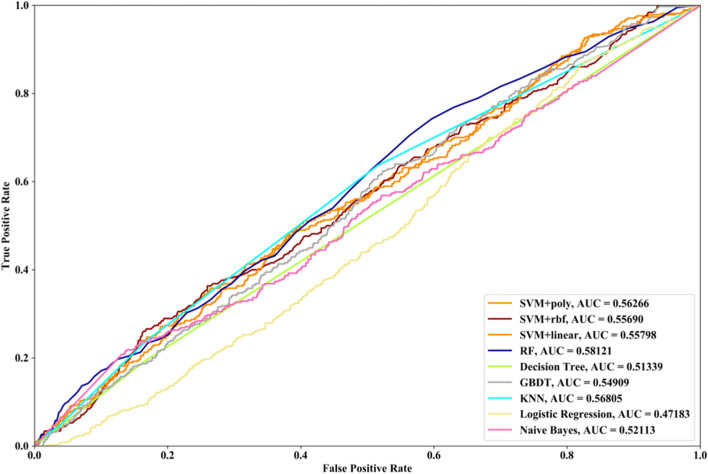
AUC and ROC performances of nine classifiers with Data3 and 10-cross validation method for GRN inference

Compared with the transcriptome data by traditional sequencing technologies, sing-cell RNA-seq data has its own internal characteristics. In this part, we compare the performances of SVM methods with different kernel functions in our proposed method. We also compare SVM with RF, NB, GBDT, DT, LR and KNN. threefold cross validation, fivefold cross validation and tenfold cross validation are utilized and the AUC results and ROC curves of nine methods with three datasets are depicted in Figs. 5, 6, 7, 8, 9, 10, 11, 12 and 13, respectively. For threefold cross validation results, SVM + rbf, NB and RF have the highest AUC values with Data1, Data2 and Data3, respectively. For fivefold cross validation results, with Data1 SVM + linear is 1.45% higher than SVM + rbf, 3% higher than SVM + poly, 14.6% higher than RF, 15.7% higher than DT, 16.3% higher than GBDT, 8.6% higher than KNN, 9.6% higher than LR and 6.1% higher than NB. With Data2, KNN has the highest AUC value, which is 0.63703. With Data3, RF also has the highest AUC value, which is 7% higher than SVM + rbf, 6.7% higher than SVM + linear, 5.4% higher than SVM + poly, 16.8% higher than DT, 6.6% higher than GBDT, 3.8% higher than KNN, 26.9% higher than LR and 15.4% higher than NB. From tenfold cross validation results, it could be seen that SVM + rbf is 0.66% higher than SVM + poly, 1.4% higher than SVM + linear, 13.7% higher than RF, 17.4% higher than DT, 14% higher than GBDT, 15.6% higher than KNN, 8.3% higher than LR and 1.0% higher than NB with Data1. With Data2 and Data3, KNN and RF have the higher AUC performances than other eight methods, respectively.

In order to compare the performances of different supervised learning methods for GRN inference obviously, we rank these nine methods according to the performances of LOOCV (Figs. 2, 3 and 4), threefold cross validation (Figs. 5, 8 and 11), fivefold cross validation (Figs. 6, 9 and 12) and tenfold cross validation (Figs. 7, 10 and 13) with three datasets. The ranking results are listed in Table 1. From Table 1, it could be clearly seen that in most cases SVM, RF and KNN methods have the highest ranking performances among nine classifiers, which show that these three methods could infer gene regulatory network more accurately. DT and LR have worse performances than other seven methods for gene regulatory network inference. Among SVM methods with three kernel functions, SVM methods with linear kernel and polynomial kernel have the higher ranking performances than SVM with rbf kernel, which prove that linear and polynomial functions are fitter to model single-cell RNA-seq data.

**Table 1:** Ranking performances of nine methods with three datasets

		SVM + poly	SVM + rbf	SVM + linear	RF	DT	GBDT	KNN	LR	NB
LOOCV	Data1	1	2	3	7	8	9	5	4	6
	Data2	4	6	3	2	8	7	1	9	5
	Data3	4	5	3	1	8	6	2	9	7
threefold cross validation	Data1	2	1	3	9	8	7	6	5	4
	Data2	4	5	3	6	8	7	2	9	1
	Data3	6	4	5	1	7	3	2	9	8
fivefold cross validation	Data1	3	2	1	7	8	9	5	6	4
	Data2	5	6	3	2	8	7	1	9	4
	Data3	3	6	5	1	8	4	2	9	7
tenfold cross validation	Data1	2	1	3	6	9	7	8	4	5
	Data2	5	6	3	4	8	7	1	9	2
	Data3	3	5	4	1	8	6	2	9	7
Average ranking		3.5	4.1	3.25	3.9	8	6.6	3.1	7.6	5

## Conclusions

In this paper, a hybrid supervised learning method based on SVM, RF, NB, GBDT, LR, DT and KNN is utilized to solve the binary classification problem of gene regulatory network inference. In SVM, three different kernel functions (linear, polynomial and radial basis function) are also utilized. Three real single-cell RNA-seq datasets from mouse and human are utilized to test these supervised learning methods. Nine supervised learning methods and one unsupervised learning method are utilized. With Data1, Data2 and Data3, in terms of AUC, SVM, KNN and RF are 0.5%-14%, 2.1%-30.3% and 2.9%-22.1% higher than other nine methods, respectively. The inference results prove that in most cases supervised learning methods (SVM, RF, NB, GBDT, LR, DT and KNN) have the better ROC and AUC performances than unsupervised learning method (GENIE3).

We also compare the performances of SVM methods with different kernel functions, RF, NB, GBDT, LR, DT and KNN further. threefold cross validation, fivefold cross validation and tenfold cross validation are utilized. The results show that in most cases SVM, RF and KNN methods have the best performances among nine classifiers. Among SVM methods with three kernel functions, SVM methods with linear kernel and polynomial kernel have the better performance than SVM with rbf kernel, which prove that linear and polynomial functions are fitter to model single-cell RNA-seq data than rbf kernel.

## Data Availability

The data used to support the findings of this study are available from the corresponding author upon request.
